# CASCADE-Cas3 enables highly efficient genome engineering in *Streptomyces* species

**DOI:** 10.1093/nar/gkaf214

**Published:** 2025-03-26

**Authors:** Christopher M Whitford, Peter Gockel, David Faurdal, Tetiana Gren, Renata Sigrist, Tilmann Weber

**Affiliations:** The Novo Nordisk Foundation Center for Biosustainability, Technical University of Denmark, 2800 Kgs. Lyngby, Denmark; The Novo Nordisk Foundation Center for Biosustainability, Technical University of Denmark, 2800 Kgs. Lyngby, Denmark; The Novo Nordisk Foundation Center for Biosustainability, Technical University of Denmark, 2800 Kgs. Lyngby, Denmark; The Novo Nordisk Foundation Center for Biosustainability, Technical University of Denmark, 2800 Kgs. Lyngby, Denmark; The Novo Nordisk Foundation Center for Biosustainability, Technical University of Denmark, 2800 Kgs. Lyngby, Denmark; The Novo Nordisk Foundation Center for Biosustainability, Technical University of Denmark, 2800 Kgs. Lyngby, Denmark

## Abstract

Type I clustered regularly interspaced short palindromic repeat (CRISPR) systems are widespread in bacteria and archaea. Compared to more widely applied type II systems, type I systems differ in the multi-effector CRISPR-associated complex for antiviral defense needed for crRNA processing and target recognition, as well as the processive nature of the hallmark nuclease Cas3. Given the widespread nature of type I systems, the processive nature of Cas3 and the recombinogenic overhangs created by Cas3, we hypothesized that CASCADE-Cas3 would be uniquely positioned to enable efficient genome engineering in streptomycetes. Here, we report a new type I based CRISPR genome engineering tool for streptomycetes. The plasmid system, called pCRISPR-Cas3, utilizes a compact type I-C CRISPR system and enables highly efficient genome engineering. pCRISPR-Cas3 outperforms pCRISPR-Cas9 and facilitates targeted and random sized deletions. Furthermore, we demonstrate its ability to effectively perform substitutions of large genomic regions such as biosynthetic gene clusters. Without additional modifications, pCRISPR-Cas3 enabled genome engineering in several *Streptomyces* species at high efficiencies.

## Graphical Abstract

**Graphical Abstract:**
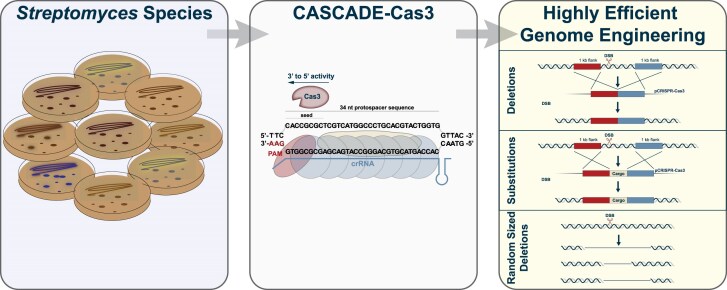

## Introduction

Streptomycetes and other filamentous actinomycetes represent some of the most gifted producers of complex specialized metabolites, with a wide diversity of applications in medicine, industry, and agriculture. Most importantly, many of the commonly used antibiotics are produced by members of the genus *Streptomyces* [1]. Many of these specialized metabolites play important ecological roles for the producer strains, e.g. to fend off competition for nutrients, communication, or to coordinate complex symbiotic relationships [2]. Due to the complex role specialized metabolites play in the natural environment, the expression of the corresponding biosynthetic gene clusters (BGCs) is tightly regulated, and natural elicitors often remain unknown. Therefore, a majority of BGCs are not readily expressed under standard laboratory conditions, hindering the identification of the encoded specialized metabolites. While classical chemical screening has resulted in the identification and characterization of high numbers of readily expressed BGCs, the advent of cheap whole genome sequencing has revealed a large untapped potential of silent BGCs, encoding the biosynthetic information for yet unknown specialized metabolites [3, 4].

To overcome these limitations and to advance the exploration of the vast biosynthetic potential of streptomycetes, several methods have been developed to activate the expression of silent BGCs. Expression of silent BGCs in a heterologous host represents one of the commonly used approaches [5, 6]. Since many actinomycetes are not (yet) genetically tractable, expression of BGCs cloned from isolated genomic DNA allows the study of BGCs independently of their native host. Furthermore, using established expression hosts streamlines experimental work as standardized methodologies can be used. Commonly used hosts for expression of heterologous BGCs include various derivatives of *S. coelicolor*, *S. albidoflavus*, *S. lividans*, *S. avermitilis*, and *S. venezuelae* [7–15].

To increase the success rate of heterologous expression of BGCs, hosts are usually engineered with focus on three aspects. First, to aid the identification of compounds in metabolomics analysis, a simplified metabolic background is desired. This can be achieved through genome reduction, with special focus on native, actively expressed BGCs, as well as those usually silent under laboratory conditions. Second, the number of integration sites can be increased to facilitate multicopy expression of the target BGC and to increase the chances of successful heterologous expression. Lastly, increasing the supply of metabolic precursors can also boost production of a matching BGC. Examples of hosts constructed using these principles are *S. albidoflavus* BE4, *S. lividans* ΔYA11, or *S. avermitilis* SUKA22 [7, 8, 14, 16].

A wide number of tools exist that can be used to achieve the above-mentioned host engineering [17]. Classically, genomic deletions were achieved through Polymerase Chain Reaction (PCR) targeting, which is based on homologous recombination and double crossovers [18]. While this method works in many *Streptomyces* strains, it requires the editing site to be cloned into a cosmid, fosmid, or bacterial artificial chromosome (BAC) and thus is labor and time intensive. Newer methods usually make use of clustered regularly interspaced short palindromic repeat (CRISPR) effectors, such as Cas9 or Cas12a. Several CRISPR-Cas9 based tools were developed for application in streptomycetes and were shown to facilitate small to medium sized deletions with good efficiencies. Common vectors include pCRISPomyces, pCRISPR-Cas9, or pKCcas9dO [19–21]. Frequently observed toxicity of Cas9, especially when strongly expressed, has since led to the development of optimized plasmids allowing tighter control of the expression of Cas9 [22]. Additionally, tools were developed utilizing alternative CRISPR-Cas systems. pKCCpf1 was developed to enable Cas12a (Cpf1) based deletions of one or two genomic loci, through Cas12a processing of its own CRISPR RNA (crRNA) array [23]. Cas12a further recognizes a T-rich protospacer adjacent motif (PAM) sequence, lowering the potential for off-target effects in the GC-rich streptomycete genome. Recently, a novel type V-F tool based on the nuclease AsCas12f1 was introduced, promising genome engineering with lower toxicity while exhibiting high efficiencies [24]. The related AsCas12j-2 nuclease was also recently utilized in streptomycetes, resulting in higher efficiencies compared to Cas12a in a previously genetically inaccessible strain [25]. Furthermore, base editing was developed for single and multiplexed inactivation of genes of interest through introduction of premature stop codons [26, 27]. While this method has many benefits, the targeted genes remain present in the genome, and off-targets are more tolerated as no double-strand breaks (DSBs) are introduced into the genome.

Type I CRISPR systems are among the most widespread CRISPR systems. The signature effector of type I systems is the nuclease Cas3, which requires the assembly of a ribonucleoprotein CRISPR-associated complex for antiviral defense (CASCADE), usually formed by Cas8, Cas7, Cas5, and the crRNA, to bind and cut the DNA [28]. Hence, CASCADE-Cas3 is a multi-effector type I CRISPR system that differs significantly from single effector type II CRISPR systems like Cas9. The most widely studied type I systems are type I-E, of which the *Escherichia coli* system has been extensively studied [29, 30]. Type I and II CRISPR systems not only differ in their overall architecture, but also in the mode of action of the signature nuclease. While Cas9 acts only as an endonuclease by introducing DSBs at the target site, Cas3 has a combined ATP-dependent nuclease-translocase activity and starts degrading DNA processively from 3′ to 5′ after cutting [31]. This results in the formation of long 5′ overhangs. CASCADE-Cas3 has previously been applied for genome engineering, but the large size of the complex has hindered widespread application using plasmid-based systems [32, 33]. Recently, Csörgő *et al.* described a compact type I-C system and its application for plasmid-based genome engineering in *E. coli*, *Pseudomonas syringae* and *Klebsiella pneumoniae* [34]. The described system requires only four genes (*cas3*, *cas5*, *cas8*, and *cas7*) to introduce efficient genomic deletions of up to half a megabase. The system recognizes a T-rich 5′-TTC-3′ PAM sequence, making it an attractive system to use in high GC organisms. Furthermore, it was shown that homology directed repair in combination with CASCADE-Cas3 introduces genomic modifications with higher efficiencies than Cas9, likely due to the recombinogenic nature of the single-stranded DNAse (ssDNAse) activity of Cas3. While the potential of genome engineering of actinomycetes using type I CRISPR systems has been discussed before, no tool based on a full type I CRISPR has yet been experimentally established [35].

Here, we present a new CASCADE-Cas3 based tool for streptomycetes, facilitating highly efficient genomic deletions and integrations based on a previously published compact type I-C CRISPR system. We adapted the system for expression in streptomycetes and integrated it into our established CRISPR plasmid platform. We demonstrate highly efficient deletions of small, mid, and large sized genomic regions, and show that pCRISPR-Cas3 works efficiently in several commonly used *Streptomyces* hosts as well as in a novel isolate. We also show that pCRISPR-Cas3 can facilitate simultaneous deletions and integrations with superior efficiencies, allowing streamlined genome engineering in even recalcitrant *Streptomyces* strains. Finally, we demonstrate the application of pCRISPR-Cas3 for streamlined genome engineering by construction of a *S. coelicolor* expression host.

## Materials and methods

### Strains and culture conditions

All work for cloning and plasmid maintenance was performed in chemically competent One Shot™ Mach1™ T1 Phage-Resistant *E. coli* cells (ThermoFisher Scientific Inc., U.S.A). Strains were cultivated on LB agar plates (10 g/l tryptone, 5 g/l yeast extract, 5 g/l sodium chloride, 15 g/l agar, to 1 l with MilliQ water) or in liquid 2× YT medium (16 g/l tryptone, 10 g/l yeast extract, 5 g/l sodium chloride, to 1 l with MilliQ water) and incubated at 37°C. If required, medium was supplemented with the appropriate antibiotics (50 ng/μl apramycin, 50 ng/μl kanamycin, and 25 ng/μl chloramphenicol). *Streptomyces* strains were cultivated at 30°C on mannitol soy flour (MS) plates (20 g/l fat reduced soy flour, 20 g/l mannitol, 20 g/l agar, 10 mM MgCl_2_, to 1 l with tap water) for spore generation. ISP2 plates (4 g/l yeast extract, 10 g/l malt extract, 4 g/l dextrose, 20 g/l agar, 333 ml tap water, and 667 ml MilliQ water) were used for clean streaking. For liquid culture, 350 ml baffled shake flasks containing 50 ml of ISP2 (supplemented with the appropriate antibiotics if needed) were inoculated from spores. All shake flask cultivations were performed in shaking incubators at 30°C and 180 rpm (311DS, Labnet International Inc., U.S.A). For actinorhodin production experiments, 24 well plates were used. Six glass beads were added into each well, and cultivations were performed in 3.6 ml of ISP2 medium. Incubations were performed at 250 rpm and 30°C. *Streptomyces* medium was supplemented with 12.5 ng/μl nalidixic acid and 50 ng/μl (solid medium) or 25 ng/μl (liquid medium) if required. For plasmid curing, plasmid harboring strains were cultivated at 40°C in non-selective ISP2 liquid medium, streaked on non-selective MS plates, and picked separately on selective and non-selective ISP2 plates for identification of plasmid-free clones.

### Cloning work

All *in silico* cloning was performed in SnapGene v6.2.1 (Dotmatics Ltd, U.S.A). Primers were ordered from IDT (Integrated DNA Technologies, U.S.A). The CASCADE-Cas3 operon was synthesized by GenScript Biotech Corporation and delivered as a plasmid. PCRs for cloning of high GC *Streptomyces* elements were performed using Q5^®^ High-Fidelity DNA Polymerase with GC Enhancer (New England BioLabs Inc., U.S.A). Colony PCRs for crRNA integrations into pCRISPR-Cas3 were performed using One*Taq^®^* 2× Master Mix with Standard Buffer Enhancer (New England BioLabs Inc., U.S.A).

Minipreps were performed using NucleoSpin^®^ Plasmid EasyPure kits (Macherey-Nagel, Germany). One percent agarose gels were run at 100 V for 20–30 min using 6× DNA Gel Loading Dye and GeneRuler 1 kb DNA Ladder (Thermo Fisher Scientific Inc., U.S.A). Both gel purifications and PCR clean-ups were performed using the NucleoSpin^®^ PCR and Gel Clean Up kit (Macherey-Nagel, Germany). DNA concentrations and purities were measured on a NanoDrop^TM^ 2000 (Thermo Fisher Scientific Inc., U.S.A). Restriction digestions were performed using Thermo Scientific^TM^ FastDigest enzymes. Differing from the manufacturer’s recommendations, the restriction digests were performed at 37°C with extended incubation times of 2 h, followed by inactivation for 10 min at 75°C. 5 U/μl T4 DNA Ligase (Thermo Fisher Scientific Inc., U.S.A) was used for ligations overnight at room temperature. Gibson assemblies were performed using NEBuilder^®^ HiFi DNA Assembly Master Mix (New England BioLabs Inc., U.S.A) following the manufacturer’s instructions. Sequence verifications were performed by Sanger sequencing using Mix2Seq overnight kits (Eurofins Genomics Germany GmbH, Germany).

### Genome mining & spacer prediction

Prediction of BGCs was performed using antiSMASH 6.0.1 [36]. Using the antiSMASH output, spacers for deletions were predicted for pCRISPR-Cas9 using CRISPy-web [37]. Spacers for pCRISPR-Cas3 were identified by searching for 5′-TTC-N_1_-N_8_-3′ sequences within the target region with no mismatches in the genome. The “find similar DNA sequences” function of SnapGene was used to identify potential off-targets with mismatches. The endogenous CRISPR system in *S. albidoflavus* J1074 was identified by searching CRISPRCasdb [38]. The presence of predicted CRISPR arrays and Cas genes was subsequently manually verified in the sequenced strain of *S. albidoflavus* J1074.

### Interspecies conjugations

Transfer of plasmid DNA into *Streptomyces* strains was performed through interspecies conjugations. Spores for conjugations were prepared as described in [39].

Homemade chemically competent or room temperature competent [40] *E. coli* ET12567 pUZ8002 [41] cells were transformed and plated on LB plates supplemented with 50 ng/μl apramycin, 25 ng/μl chloramphenicol, and 50 ng/μl kanamycin. All transformants were washed off the plate with 4 ml LB medium, and 5 ml LB overnight cultures were inoculated with 50 μl washed cels. Two millilitres of the overnight cultures were harvested the next day and washed twice at 2000× *g* with 1 ml of fresh LB medium. Five hundred microlitre of resuspended *E. coli* ET12567 pUZ8002 cells were then mixed with 500 μl of filtered spore suspension and spread on MS + 10 mM MgCl_2_ plates. The next day, overlays were performed with 1 ml of ddH_2_O and 5 μl of 50 mg/ml apramycin. Exconjugants were picked using sterile wooden toothpicks and transferred to selective ISP2 plates supplemented with nalidixic acid.

### *Streptomyces* colony PCRs

For colony PCRs on *Streptomyces* colonies, pieces of the colonies were scraped off the plates and transferred to PCR tubes containing 50 μl of 10% DMSO. The tubes were sealed and boiled for 10 min at 99°C, transferred to dry ice for 20 min, and then again boiled for 10 min. The entire process was repeated, and the tubes then spun down to separate the debris from the supernatant. One microlitre of the supernatant was used as template for colony PCRs. *Streptomyces* colony PCRs were performed using Q5^®^ High-Fidelity DNA Polymerase with GC Enhancer (New England BioLabs Inc., U.S.A).

### Whole genome sequencing & bioinformatic analysis

Genomic DNA was isolated from *Streptomyces* plates or liquid cultures using the DNeasy PowerLyzer PowerSoil kit (Qiagen, Germany). The bead beating was performed using a TissueLyser LT (Qiagen, Germany) at default settings for 7 min. Quality control was performed using a NanoDrop^TM^ 2000 for purity and Qubit 4 Fluorometer for concentration measurements (Thermo Fisher Scientific Inc., U.S.A). Genomic DNA was run on 0.8% agarose gels to assess fragmentation. Illumina sequencing was performed by Novogene Co., Ltd (Bejing, China). Libraries were prepared using the NEB Next® UltraTM DNA Library Prep kit (New England Biolabs, U.S.A) with a target insert size of 350 nt and six PCR cycles. trimgalore (https://github.com/FelixKrueger/TrimGalore) and breseq 0.33.2 [42] were used for read trimming and data analysis of illumina sequencing data. The following commands were used:


\begin{eqnarray*}
&&{trim\_galore{\mathrm{ }} - j\,\, 8{\mathrm{ }} - - length{\mathrm{ }} 100\ - o\,\,illumina{\mathrm{ }}} \\ && \quad {- - paired{\mathrm{ }} - - quality{\mathrm{ }}\,\, 20{\mathrm{ }} - - fastqc{\mathrm{ }} - - gzip{\mathrm{ }}file\left( s \right)}\\ && {breseq{\mathrm{ }} - r{\mathrm{ }}\left( {full{\mathrm{ }}\,\, genetic{\mathrm{ }} \,\, background{\mathrm{ }}\,\, reference}.gb \right)}\\ &&{trimmed\_1.fq.gz{\mathrm{ }} \,\, trimmed2.fq.gz{\mathrm{ }} \,\, - j\,\,{\mathrm{ } }12}
\end{eqnarray*}

Using samtools [43], the read coverage was extracted from the the breseq output as follows:


\begin{eqnarray*}
samtools{\mathrm{ }} \,\, depth{\mathrm{ }}\,\, - a{\mathrm{ }} \,\, sample.bam{\mathrm{ }} >{\mathrm{ }}sample.coverage
\end{eqnarray*}

Oxford Nanopore sequencing was performed as described by Alvarez-Arevalo *et al.* [44], but using the gDNA isolation method described above. Basecalling was performed using Guppy (5.0.17 + 99baa5b, client-server API version 7.0.0 or 6.3.8 + d9e0f64, client-server API 13.0.0) applying the high-accuracy model and excluding reads <1 kb.

Minimap2 2.18 [45] was used to map reads and samtools was used to extract the reads as follows:


\begin{eqnarray*}
\begin{array}{@{}*{1}{l}@{}} {minimap2{\mathrm{ }}\,\, - a{\mathrm{ }}\,\, reference.fa{\mathrm{ }}\,\, reads.fastq.gz{\mathrm{ }} >{\mathrm{ }}mapping.sam}\\ {samtools{\mathrm{ }}\,\, sort{\mathrm{ }}\,\, mapping.sam{\mathrm{ }} > {\mathrm{ }}mapping.sam.sort.bam}\\ {samtools{\mathrm{ }}\,\, depth{\mathrm{ }}\,\, - a{\mathrm{ }}\,\, mapping.sam.sort.bam{\mathrm{ }} > {\mathrm{ }}sample.coverage} \end{array}
\end{eqnarray*}

Mappings were visualized using Artemis [46]. For plotting, the regions of interest were extracted from the coverage files and plotted in Prism 9 (GraphPad Software, U.S.A). *De novo* assemblies were performed using flye 2.9 [47]. Assembly graphs were visualized using bandage 0.8.1 [48].

Sequencing of BAC integration strains was performed using the rapid barcoding kit SQK-RBK114.96 (Oxford Nanopore Technologies, U.K.) and sequenced on an R10.4.1 MinION flowcell (Oxford Nanopore Technologies, U.K.). The data were basecalled using Guppy (7.3.11 + 0112dde09) applying the super-accuracy model. Reads were mapped as described in the previous section. All reads that mapped successfully to the BAC sequence were mapped against *S. coelicolor* CW6 E2 to determine insertion sites and copy number by manual inspection. Results were confirmed by mapping the reads against an *in silico* reference of *S. coelicolor* CW6::act.

### Cell dry weight measurements

The endpoint cell dry weight was measured during harvesting of cultures. Two millilitre tubes were dried overnight at 60°C. For each strain, three replicates were measured, and individual tubes were weighed and numbered. Two millilitres of cell culture was added, spun down at 21 000× *g*, and washed with ddH_2_O. After removing all supernatant, the tubes were then dried overnight at 60°C and measured the next day on a Sartorius Qunitix scale (1 mg).

### Actinorhodin measurements

For actinorhodin quantification, 2 ml of the shake flask cultures was harvested by centrifugation for 10 min at 21 000× *g*. The supernatant was transferred to a new tube, and 100 μl was transferred to F-bottom, clear, 96 well microplates (Greiner Bio-One International GmbH, Austria). 50 μl of 3 M NaOH was added to each well and carefully mixed by pipetting. The samples were then measured in a BioTek EPOCH2 microplate reader (Agilent Technologies, Inc.) at 640 nm.

### Computational scripts

The spacer identification Jupyter Notebook was written in Python 3.8.16 and uses pandas (v. 1.5.3), Biopython (v.1.81), and the Python regular expressions module. The notebook is available on GitHub (https://github.com/NBChub/Type1_Protospacers).

## Results

### Distribution of type I CRISPR systems in streptomycetes

**Figure 1.**
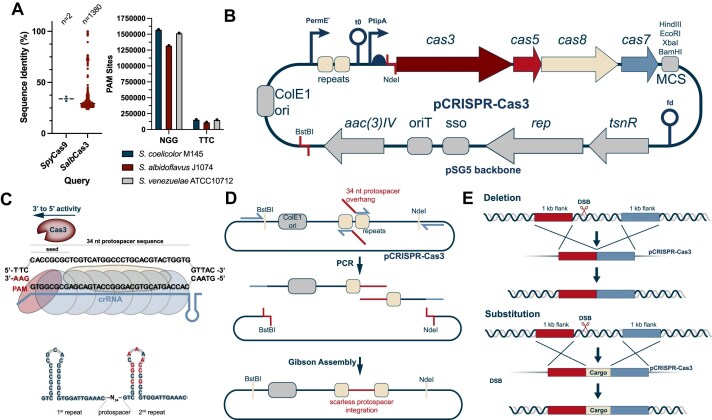
pCRISPR-Cas3 enables streamlined genome engineering of streptomycetes. (**A**) Distribution of type I and type II CRISPR systems in streptomycetes and the number of PAM sites identified in selected streptomycetes for both systems. Spycas9 and cas3 from *S. albidoflavus* were used as references. The BLAST search was run on all high quality publicly available *Streptomyces* genomes (*n* = 2401). Type I CRISPR systems appear to be much wider distributed than type II CRISPR systems in streptomycetes. However, only 108 hits with a sequence similarity above 50% were identified. An order of magnitude fewer PAM sites were identified in three selected streptomycetes for the CASCADE-Cas3 PAM compared to the NGG Cas9 PAM, highlighting the much lower number of potential off-target sites. (**B**) Plasmid map of pCRISPR-Cas3. The plasmid is based on the pSG5 replicon and carries the codon optimized type I-C minimal CASCADE under control of the inducible tipA promoter. All elements of the backbone are highlighted in gray. The crRNA is cloned between to repeats in the chromosomal RNA (cRNA) cassette, which is controlled by the constitutive ermE* promoter. Repair templates are cloned into the multiple cloning site (MCS) on the backbone of the plasmid. (**C**) The second repeat has a modified sequence to prevent recombination between the two repeats. The CASCADE complex comprised of Cas5, Cas8, and Cas7 units binds the target sequence and recruits Cas3. Cas3 has a 3′–5′ helicase nuclease activity, resulting in directionally biased deletions. (**D**) cRNAs are cloned between two repeats, posing some challenges due to sequence homologies. Since type IIS restriction enzymes cannot be used in high GC *Streptomyces* plasmids, a PCR and Gibson Assembly based cloning approach was established, allowing cloning of cRNAs with high efficiencies. (**E**) pCRISPR-Cas3 can be used for targeted deletions of large genomic regions, or for substitutions of such with a specified cargo. It can also be used for random sized deletion experiments.

Type I CRISPR systems are widespread in bacteria and archaea. However, only a few systems from *Streptomyces* have been characterized and described in detail so far [49]. To investigate the distribution of type I CRISPR systems versus type II CRISPR systems in streptomycetes, we performed BLAST searches against 2401 high quality publicly available *Streptomyces* genomes. The amino acid sequence of Cas9 from *Streptococcus pyogenes* and of Cas3 of the type I-E CRISPR system of *S. albidoflavus* (previously *albus*) J1074 were used as queries, as these are the hallmark nucleases from the two CRISPR types. Type I CRISPR systems appear to be more widespread in *Streptomyces* compared to type II CRISPR systems. Only two hits were obtained for *Spy*Cas9, while *Salb*Cas3 returned almost 1400 hits (Fig. 1). Of these, over 100 had a sequence similarity >50% (Supplementary Table S1). Cas3 acts as a nuclease-translocase with a N-terminal HD phosphohydrolase and C-terminal helicase domain [50]. To obtain a more granular view of the distribution of type I systems in streptomycetes, and to reduce the number of false positive hits due to similarities to helicases, we performed another search with cblaster. This enabled the search for clustered homologous sequences [51]. Using the CASCADE from *S. albidoflavus* J1074 as input, we identified 472 strains carrying the entire CASCADE, comprising of *cas3*, *cas8*, *cas11*, *cas7*, *cas5*, *and cas6* (Supplementary Table S2).

Given the high abundance of type I CRISPR systems in streptomycetes, we wanted to investigate whether these systems might be intrinsically better suited for genome engineering due to lower potential toxicity in comparison to type II systems. This could be attributed to the low GC PAM of type I CRISPR systems, which minimizes the number of potential endogenous target sites in case of acquisition of self-targeting spacers [52]. Furthermore, even in the event of self-targeting, the single stranded recombinogenic overhangs produced by Cas3 may allow easier repair of the DSB, increasing the chances for homology directed repair using microhomologies or larger homologous sequences.

### Construction of pCRISPR-Cas3, a plasmid based compact CASCADE-Cas3 system

The previously characterized and utilized type I-C CRISPR system from *Pseudomonas aeruginosa* consists of only four genes, *cas3*, *cas5*, *cas8*, and *cas7*, totaling 5.6 kb [34]. This allows plasmid-based expression of the system and use across various species. All four genes are arranged in an operon and can hence be expressed from a single promoter. Given the differences in both codon usage and GC content, the previously published operon was codon optimized and synthesized. For codon optimization, the *S. coelicolor* codon usage table was used, as such optimized constructs have previously been successfully expressed in a wide variety of different streptomycetes [39]. The upstream region of each *cas* gene was designed with a canonical ribosome binding site (RBS) sequence (GGAGG or GGAGC). The 5.6 kb fragment was subsequently synthesized and delivered as a cloned plasmid. For expression in streptomycetes, our established CRISPR platform based on the pSG5 replicon was used (Fig. 1B). The Cas9 cassette from pCRISPR-Cas9 was removed by digestion with NdeI and HindIII and replaced by the synthesized CASCADE-Cas3 operon using Gibson Assembly. The Cas9 gRNA expression cassette was then replaced by a double-stranded DNA fragment containing the Cas3 repeats following digestion of the plasmid with NcoI and SnaBI and subsequent Gibson Assembly. The second repeat was modified as described by Csörgő *et al.* to prevent recombination events between the two repeats (Fig 1C). The resulting plasmid was named pCRISPR-Cas3 in accordance with our previous plasmid nomenclature. Expression of CASCADE-Cas3 is controlled by the thiostrepton inducible *tipA* promoter. As with pCRISPR-Cas9 and CRISPR-BEST, the basal expression level of the *tipA* promoter is usually sufficient to achieve successful editing in most streptomycetes. While homologues of *tipA* are widespread in streptomycetes, to allow expression from the tipA promoter in strains lacking it, *tipA* might need to be coexpressed from the backbone.

Given the repetitive nature of the Cas3 CRISPR repeats (Fig. 1C), cloning spacer sequences in between these proved to be very challenging. Attempts to clone spacer sequences using ssDNA oligo bridging by digestion of pCRISPR-Cas3 with NcoI (cutting between the two repeats) failed repeatedly. Given that common type IIS restriction enzymes such as BsaI and BstBI, commonly used for such cloning scenarios, cannot be used in high GC plasmids due to their omnipresent recognition sequences, further complicated this cloning challenge. To achieve efficient cloning of user defined spacer sequences between the CRISPR repeats, we had to design an alternative cloning strategy (Fig. 1D). Using two fixed primers binding up and downstream from the spacer integration site, two PCRs are set up using primers binding each of the repeats and carrying the desired 34 nt spacer sequence in the overhangs. Digestion of pCRISPR-Cas3 with BstBI and NdeI removes the region of the plasmid containing the spacer integration site, as well as the origin of replication (Fig. 1B), ensuring that only correctly assembled plasmids can replicate. Following purification of the PCR fragments and the backbone, a standard Gibson Assembly is performed. Using this cloning method, we frequently achieved over 90% cloning efficiencies (Supplementary Fig. S1).

### pCRISPR-Cas3 introduces highly efficient deletions with single nucleotide precision in *S. coelicolor*

**Figure 2.**
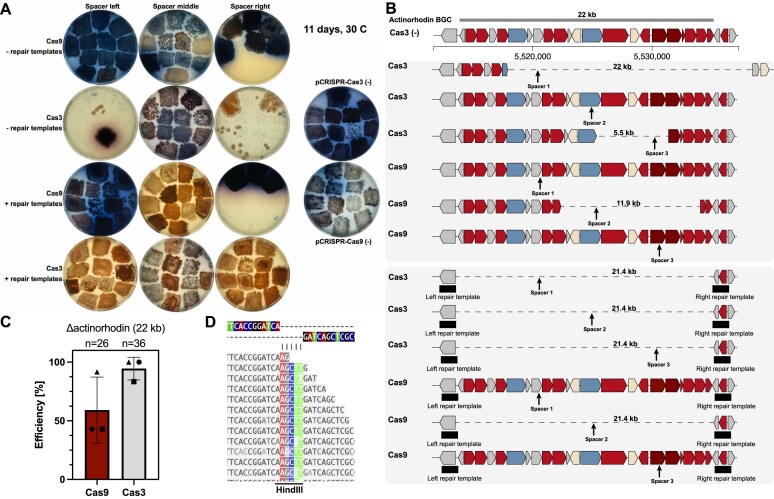
pCRISPR-Cas3 introduces genomic deletions with higher efficiencies than pCRISPR-Cas9. (**A**) Plate pictures of *S. coelicolor* mutants harboring pCRISPR-Cas9 or pCRISPR-Cas3 with spacers targeting the actinorhodin BGC in three different locations, and with or without repair templates. pCRISPR-Cas3 displayed higher toxicity without repair templates but resulted in more exconjugants overall and more with the desired red phenotype once repair templates were provided. (**B**) Representation of sequencing results of selected colonies, both for pCRISPR-Cas3 and pCRISPR-Cas9 with and without repair templates. Both pCRISPR-Cas3 and pCRISPR-Cas9 introduced random sized deletions without repair templates. With repair templates, precise deletions were observed for both pCRISPR-Cas3 and pCRISPR-Cas9. The spacers 1 for Cas9 and CASCADE-Cas3 targeted the left flank of the BGC, spacers 2 the middle, and spacers 3 the right flank. (**C**) Efficiencies for actinorhodin deletions with pCRISPR-Cas9 and pCRISPR-Cas3. For pCRISPR-Cas3, the efficiencies were consistently high (>80%), while with pCRISPR-Cas9 the observed efficiencies were highly sgRNA dependent. Circles correspond to data for spacers 1, triangles for spacers 2, and rectangles for spacers 3. (**D**) Read alignments for the junction site of the two homologous flanks. A HindIII site was integrated, demonstrating that the DSB was repaired using the repair templates cloned into pCRISPR-Cas3 using HindIII. Shown in panel (C) are the means ± standard deviations of three deletion experiments targeting the actinorhodin region with three different spacers.

Given the processive nature of Cas3, the primary applications of interest of pCRISPR-Cas3 are genomic deletions or substitutions (Fig. 1E). To demonstrate the application of pCRISPR-Cas3 for genome engineering in streptomycetes, we attempted to delete the well-characterized 22 kb actinorhodin BGC in *S. coelicolor*. Deletion of the actinorhodin BGC leads to a clear red phenotype in *S. coelicolor*, resulting from expression of the undecylprodigiosin BGC and enabling phenotypical screening for deletion mutants (Fig. 2A). Three different spacers were selected, in the middle and towards each edge of the deletion region, to investigate potential differences in editing outcomes based on spacer positions relative to the repair templates (Fig. 2B). Spacers for pCRISPR-Cas3 were manually designed based on PAM + seed sequence queries within the deletion region, followed by genome wide queries using both the seed and whole length sequences of putative spacers to identify the most specific candidates. In *S. coelicolor*, 158 341 5′-TTC-3′ PAM sequences were found, averaging one PAM for every 54.7 bp. In contrast, 1 574 641 5′-NGG-3′ PAM sequences were found for Cas9, averaging one for every 5.5 bp, and highlighting the presence of up to an order of magnitude more potential off-target sites (Fig. 1A). Two flanking regions of 1 kb were selected as repair templates and cloned into the MCS of pCRISPR-Cas3 using restriction cloning as described in the “Materials and methods” section. To compare editing outcomes to those achieved with Cas9, the same repair templates were cloned into pCRISPR-Cas9, and three different single guide RNAs (sgRNAs) mirroring the positions of the spacers selected for pCRISPR-Cas3 were designed. In parallel, all experiments with pCRISPR-Cas3 and pCRISPR-Cas9 were also performed without repair templates.

Stark differences were observed between the different editing configurations. pCRISPR-Cas9 without repair templates failed to produce the distinct red phenotype, and with the sgRNA located in the right side of the deletion region, only a handful of viable exconjugants were obtained on selective medium (Fig. 2A). Given that the pCRISPR-Cas9 plasmid without ScaLigD was used, higher host toxicity was expected. As shown previously, co-expression of ScaLigD can greatly enhance non-homologous end joining and reduce the toxicity in *S. coelicolor* [20]. pCRISPR-Cas3 without repair templates resulted in greatly varying outcomes, depending on the used spacer. Both spacers close to the edges of the deletion region appeared to result in high host toxicity. However, both clones obtained using the right spacer had a weak red phenotype. pCRISPR-Cas3 without repair templates and the spacer in the middle of the deletion region did not result in observable loss in viability, but also did not produce a clear red phenotype. Clear deletion phenotypes were obtained with pCRISPR-Cas9 with repair templates and the sgRNA target located in the middle of the deletion region. However, with the other two sgRNAs, either no clear deletion phenotypes or only a small number of viable exconjugants were obtained, which did not have the desired phenotype. The observed toxicities of pCRISPR-Cas9 might be predominantly spacer specific. However, recent studies have shown that binding to off-target sites might be much more extensive, stabilized by as little as a few nucleotides of homology [53]. Using pCRISPR-Cas3 together with repair templates, clear deletion phenotypes were obtained for all spacers. With spacer targets located towards the edges of the deletion region, clear deletion phenotypes were obtained for all picked exconjugants. Only 5 out of 12 exconjugants showed the expected phenotype when the spacer target was located in the middle of the deletion region, with the remaining seven having a grayish phenotype. Interestingly, these results appeared to be opposite to pCRISPR-Cas9, which performed best with the spacer target located in the middle of the deletion region.

Phenotypical screening revealed an apparent superiority of pCRISPR-Cas3 compared to pCRISPR-Cas9, as all three spacer configurations resulted in the expected deletion phenotype with high efficiencies. However, given the complex lifecycle and regulatory networks of streptomycetes, phenotypic assays can be misleading. For example, the greyish colonies, observed for pCRISPR-Cas3 with repair templates and the spacer located in the middle of the deletion region, appeared to turn slightly red at the edges of the colonies, hinting at potentially delayed onset of undecylprodigiosin production compared to the other colonies. Therefore, to verify the observations from the first experiment, and to obtain more precise and reliable efficiencies for pCRISPR-Cas9 and pCRISPR-Cas3, the experiments with spacers and repair templates were repeated. This time, conjugations were performed at larger scales to obtain more viable clones for pCRISPR-Cas9 with the spacer targeting the right flank of the deletion region. The deletion efficiencies were analyzed by performing colony PCRs on the targeted region with a primer binding inside of the repair template, and one binding outside of the repair template in the neighboring genomic sequence (Supplementary Fig. S2). For pCRISPR-Cas9, average deletion efficiencies of 60% were obtained. These varied greatly depending on which spacer was used. For pCRISPR-Cas3, an average efficiency of 94% was obtained (Fig. 2C). The obtained efficiencies for pCRISPR-Cas3 were consistently high for all spacers, suggesting a lower dependency on the spacer sequence and location to achieve efficient deletions.

To verify the PCR results, Illumina based whole genome sequencing was performed for one colony of each configuration (Fig. 2B). Random sized deletions were observed for both pCRISPR-Cas9 and pCRISPR-Cas3 when no repair template was provided. For pCRISPR-Cas3, the observed random sized deletions were 5.5 and 22 kb in size, and for pCRISPR-Cas9 11.9 kb. No random sized deletions were observed for pCRISPR-Cas3 with the spacer targeting the middle of the deletion region, and none for pCRISPR-Cas9 when the sgRNAs targeting the edges of the deletion region were used. Sequencing of repair template containing configurations showed that pCRISPR-Cas3 successfully introduced the designed mutations with single nucleotide precision for all tested configurations (Fig. 2D). For pCRISPR-Cas9, only the configuration using the sgRNA targeting the middle of the deletion region resulted in successful deletion of the designed region.

As pCRISPR-Cas3 was able to introduce the designed deletions with robust efficiencies and single nucleotide precision, we next investigated whether pCRISPR-Cas3 could be used to install targeted deletions in other *Streptomyces* species.

### Genomic deletions were achieved using pCRISPR-Cas3 in *S. venezuelae*, *S. albidoflavus*,and *Streptomyces* sp. NBC01270

**Figure 3.**
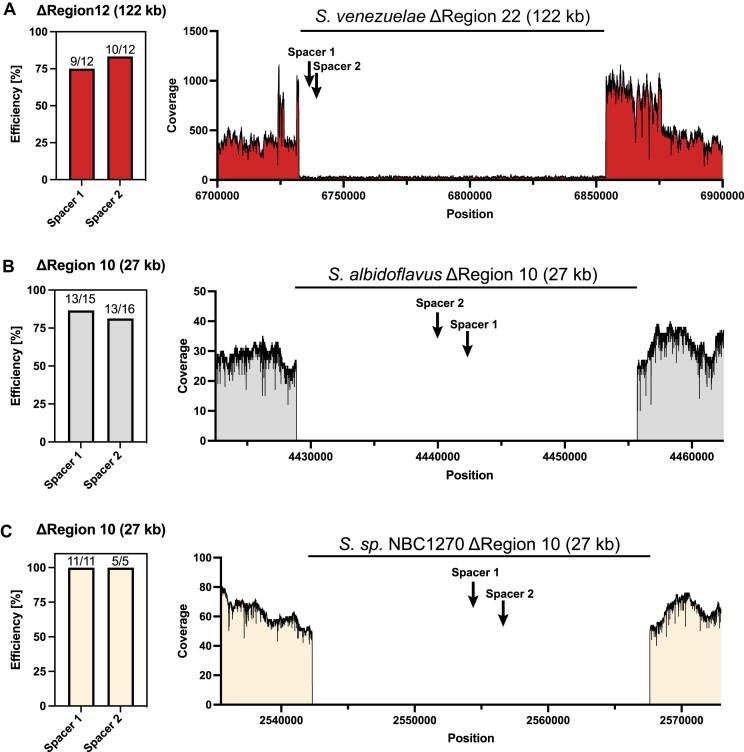
Installation of highly efficient deletions in *S. venezuelae* (**A**), *S. albidoflavus* J1074 (**B**), and *Streptomyces* sp. NBC1270 (**C**). In *S. venezuelae*, antiSMASH region 22 (122 kb) was deleted, encoding a dense accumulation of BGCs. In *S. albidoflavus* J1074 and *S*.sp. NBC1270, the 27 kb fluostatin-like BGC encoded by region 10 was deleted. In all strains, deletions were achieved with high efficiencies, ranging from 75% to 100% of all screened colonies.

Given the successful demonstration in *S. coelicolor*, we next attempted to install targeted deletions in other strains of interest. Therefore, we selected *S. venezuelae* (ATCC 10712), an emerging production host and model species, *S. albidoflavus* J1074, a well-established host for expression of heterologous BGCs, and *Streptomyces* sp. NBC1270, an isolate from the NBC strain collection closely related to *S. albidoflavus* J1074. For all experiments, repair templates of 1 kb were used flanking the target region, and deletions were attempted with two different spacer sequences (Fig. 3).

In *S. venezuelae* ATCC 10712, region 22 predicted by antiSMASH 6.0.1 was selected as a target for demonstration, given its large size of 122 kb and the high density of putative BGCs. Both repair template and spacer combinations introduced the desired deletion with high efficiencies, with 9 out of 12 and 10 out of 12 exconjugants carrying the designed mutation, respectively (Fig. 3A). The PCR based screening results (Supplementary Fig. S5) were further verified by whole genome sequencing, showing a clearly defined deletion as indicated by the drop in coverage of region 22. Importantly, spacers 1 and 2 targeted different strands, demonstrating that highly efficient deletion can be installed independently of the spacer target strand.

*S. albidoflavus* J1074 and *S*.sp. NBC1270 are closely related and share the majority of BGCs as predicted by antiSMASH 6.0.1. In *S. albidoflavus* J1074 and *Streptomyces*sp. NBC1270, we targeted the 32.4 kb fluostatin-like lanthipeptide BGC, which is shared between the two strains. Using the same plasmids in both strains, precise deletions were obtained with both spacers at high efficiencies. In *S. albidoflavus* J1074, based on PCR screening, 87% and 81% of screened colonies carried the desired deletion when targeted with spacers 1 and 2, respectively (Fig. 3B and Supplementary Fig. S5). In *S*. sp. NBC1270, 100% of all colonies resulted in bands indicating successful deletion of the BGC (Fig. 3C). To verify these PCR based results, we sequenced PCR-positive colonies using Oxford Nanopore sequencing. Mapping of the reads against the respective reference genome verified precise deletions in both *S. albidoflavus* J1074 and *S*. sp. NBC1270.

We further attempted to delete a dense accumulation of three BGCs for production of antimycin, candicidin, and flaviolin located at the far terminal chromosomal end in both *S. albidoflavus* J1074 and *S*.sp. NBC1270. Given the total size of 318 kb, this large region was also interesting to investigate the possibility of large single step deletions. Following several failed attempts to obtain PCR bands indicating successful introduction of the designed chromosomal deletion, some of the colonies for which no PCR bands were obtained were sequenced using Oxford Nanopore sequencing. In both *S. albidoflavus* J1074 and *S*.sp. NBC1270, targeting of the far chromosomal end resulted in unspecific deletions ∼380 and 140 kb in size, respectively. In *S. albidoflavus* J1074, this resulted in deletion of antiSMASH regions 19–23. Targeting of the same region with pCRISPR-Cas9 also resulted in extended deletions, suggesting that this was a general problem resulting from introduction of DSBs at the ends of the chromosomal arms [54]. Interestingly, targeting the same region in *S*.sp. NCB1270 using pCRISPR-Cas9 resulted in loss of the entire chromosomal end, as no reads mapped against the terminally inverted repeats (Supplementary Fig. S3). A big spike in coverage was observed just at the end of the deletion region, suggesting that this 33 kb sequence stretch was replicated around 12 times to prevent continuous degradation of the chromosomal end. De novo assembly of the genome and subsequent visualization of the assembly graph further confirmed this hypothesis (Supplementary Fig. S4). Both strains did not display any obvious growth defects on plates, and all desired BGCs were deleted in *S. albidoflavus* J1074, despite the unspecific nature of the deletion.

These results clearly demonstrate how pCRISPR-Cas3 can be used to introduce both random sized deletions and precise deletions in several important *Streptomyces* species. Our results further highlight that pCRISPR-Cas3 can enable efficient genome engineering even in non-model *Streptomyces* species.

### Simultaneous deletions and integrations can be achieved through modification of the repair templates

**Figure 4.**
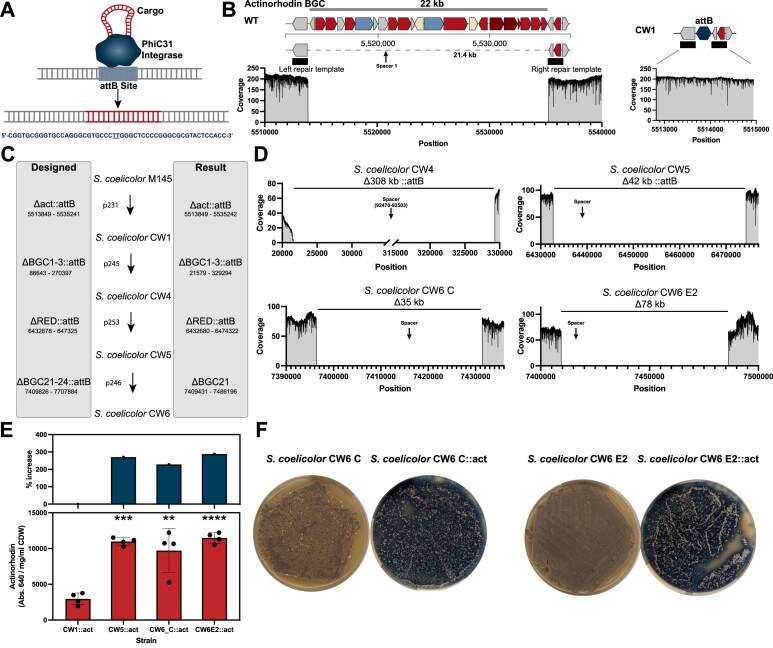
Simultaneous deletions and integrations enable streamlined genome engineering. (**A**) The PhiC31 *Streptomyces* integrase integrates cargo DNA into target *attB* sites. The consensus *attB* site from *S. coelicolor* is 51 bp long and features a central TT sequence where the cargo is integrated. (**B**) Substitution of the entire actinorhodin BGC with an additional *attB* site. The *attB* site was cloned between the repair templates. Coverage plots of mappings of ONT data against the wild type and the in silico generated substitution strain reveal precise genome engineering. (**C**) pCRISPR-Cas3 was used for construction of a *S. coelicolor* expression host using both targeted and semi-targeted deletions and substitutions. (**D**) Oxford Nanopore sequencing results for all deletions based on minimap2 mappings to the reference genome. (**E**) The final strains *S. coelicolor* CW5 and CW6 both displayed >200% increase in actinorhodin production compared to the base strain *S. coelicolor* CW1 upon integration of an actinorhodin BGC BAC. (**F**) Phenotypes of *S. coelicolor* CW6 C and E2 without and with actinorhodin integrations. Shown in panel (E) are the means ± standard deviations of four biological replicates. Significance was tested using unpaired two tailed *t*-tests, where **P < .01, ***P < .001, and ****P < .0001.

Most sophisticated engineered *Streptomyces* hosts have a reduced metabolic background, achieved through deletion of readily expressed BGCs, and added integration sites for multicopy integrations or site directed targeted integrations with different integrases [7, 14, 55]. Consequently, we wanted to demonstrate how pCRISPR-Cas3 could be used to simultaneously delete genomic regions, and install additional integration sites. The *Streptomyces* bacteriophage PhiC31 integrase is a well-established system for integration of heterologous sequences in various *Streptomyces* species [56, 57] (Fig. 4A). The consensus PhiC31 attachment site (*attB*) from *S. coelicolor* [58] was chosen as cargo and cloned in between the two repair template flanks. As a proof of concept, we modified plasmid p129, previously used to delete the actinorhodin BGC in *S. coelicolor*, to carry the *attB* site between the two homologous repair template arms. The obtained sequencing reads mapped perfectly against the *in silico* modified reference sequence (Fig. 4B).

### Cas3-based genome engineering enables rapid host construction in *S. coelicolor*

Motivated by these results, we wanted to expand on this approach and demonstrate how pCRISPR-Cas3 can enable streamlined genome engineering of *Streptomyces* hosts. *S. coelicolor* has previously been engineered as a host for heterologous expression of BGCs, and a number of derivatives were constructed in the process [59]. The most widely used ones, *S. coelicolor* M1152 and M1154 are both quadruplicate deletion strains (*Δact Δred Δcpk Δcda*) with varying additional mutations. To demonstrate how pCRISPR-Cas3 can be used for streamlined genome engineering experiments, we attempted to perform simultaneous genome reduction experiments and integrations of additional *attB* sites. It was previously shown that the integration site of BGCs can have great effects on product titers, and that integrations in the chromosomal arms, where the BGC density is bigger, generally result in higher titers [60]. Therefore, integration of heterologous BGCs into *attB* sites, which substituted native BGCs is likely to result in desirable production phenotypes.

Based on the *S. coelicolor* M145 Δact::*attB* strain, from here on referred to as *S. coelicolor* CW1, several rounds of pCRISPR-Cas3 mediated genome engineering experiments were performed (Fig. 4C). Given the processive nature of the Cas3 nuclease, we utilized a combination of targeted and semi-targeted deletions. As observed in *S. albidoflavus* J1074 and *S*. sp. NBC1270, Cas3 can install deletions extending beyond the target region if larger or instable regions are targeted. We exploited this ability to not just delete single BGCs, but to also target larger regions with a high density of BGCs. The selected chromosomal regions contained antiSMASH regions 1–3 and 21–24, and encode several well characterized BGCs, including those for isorenieratene, hopene, and arsono-polyketide. Targeting of regions 1–3 using p245 (supplementary information, plasmids and primers) resulted in a deletion larger than intended, similar to what was observed in *S. albidoflavus* J1074 and *S*. sp. NBC1270 (Fig. 4D). Nonetheless, the deleted region was substituted by an additional *attB* site, suggesting that the repair templates must have been used during the recombination event. The resulting strain with four deleted BGCs and two additional *attB* sites was named *S. coelicolor* CW4 and used as the basis for the next round of genome engineering. Using plasmid p253, the undecylprodigiosin BGC was deleted and substituted by a third additional *attB* site. The deletion was very precise and varied only by a few nucleotides from the *in silico* designed deletion, again highlighting how pCRISPR-Cas3 can be used for both highly precise as well as semi-targeted random sized deletions. The new strain was named *S. coelicolor* CW5 and used for the final round of genome engineering, targeting regions 21–23 with plasmid p246. The resulting deletion was smaller in size than designed and resulted in only the deletion of region 21. Additionally, no *attB* site was integrated, suggesting that this deletion was fully unspecific. The final strain, named *S. coelicolor* CW6 was reduced by 6 BGCs and carries a total of four *attB* sites, three of which were integrated using pCRISPR-Cas3 (Fig. 4E). In total, the genome was reduced by around 450 kb, corresponding to a genome reduction of ∼5%. In addition to the pCRISPR-Cas3 induced deletions, we observed several putative transposition events. These were relatively stable across all sequenced strains, indicating that the majority of these came already from the parental *S. coelicolor* M145 strain (Supplementary Table S3).

To test the constructed *S. coelicolor* CW strains, we introduced the actinorhodin BGC encoded on an integrative plasmid via conjugation into all strains. *S. coelicolor* CW1 was used as the base strain, and *S. coelicolor* CW5 and *S. coelicolor* CW6 as final strains. For *S. coelicolor* CW6, two clones were tested. In addition to *S. coelicolor* CW6 E2, in which the deletion size in region 21 was 76.7 kb, we also tested *S. coelicolor* CW6 C, where the deletion was only 35 kb in size (Fig. 4D). After conjugation, four biological replicates were selected of each strain and cultivated in ISP2 medium supplemented with apramycin for one week in 24 well plate as described in the “Materials and methods” section. The actinorhodin production was measured in the supernatant at 640 nm and normalized with the cell dry weight of the respective culture. *S. coelicolor* CW5 and CW6 strains all produced significantly more actinorhodin compared to the base strain *S. coelicolor* CW1 (Fig. 4E). For *S. coelicolor* CW5, the specific product yield rose from 2956 Abs._640nm_ /mg ml^−1^ CDW to 10974 Abs._640nm_ /mg ml^−1^ CDW, an increase of 271%. For *S. coelicolor* CW6 C, the specific production was 9713 Abs._640nm_ /mg ml^−1^ CDW, corresponding to an increase of 228% compared to *S. coelicolor* CW1. Finally, for *S. coelicolor* CW6 E2, a specific actinorhodin production of 11480 Abs._640nm_ /mg ml^−1^ CDW was measured, an increase of 288% over *S. coelicolor* CW1. All hosts also produced actinorhodin on plates, highlighting robust expression independent of cultivation method (Fig. 4F). These results highlight how the streamlined deletion of BGCs and integration of additional *attB* sites can be used to construct potential new expression hosts.

### Analysis of BAC integration frequency and genome stability

To analyze the number of integrations as well as the impact of multiple integrations on genome stability, an additional set of experiments was performed using strain *S. coelicolor* CW6 E2 with the actinorhodin BGC reintegrated. Spores were used to inoculate precultures in apramycin supplemented ISP2 medium. After 5 days, non-selective main cultures were inoculated from the preculture, and samples were taken after 48 h. Genomic DNA was isolated from spores and the 48 h samples of two biological replicates. Whole genome sequencing using Oxford Nanopore revealed BAC integrations into the native phiC31 *attB* site, as well as the Δact::*attB* and ΔRED::*attB* loci in *S. coelicolor* CW6::act. Comparison of the spore data with the endpoint data showed that the integrations were stable across all conditions and were not lost during cultivation in non-selective medium (Supplementary Table S4). Integrations into the ΔBGC1-3::*attB* locus were not observed, potentially due to genomic instabilities caused by integrations in such proximity to the chromosomal terminus. Integrations of multiple copies of the actinorhodin BGC also appeared to have no major influence on genomic stability. The only observed unintended modifications were transposition events, which were observed already in previous versions of the strain and are therefore unrelated to the BAC integrations (Supplementary Table S3).

### Automated identification and filtering of compatible spacer sequences

Given that the majority of existing computational tools do not support type I/type I-C CRISPR systems, we developed a simple interactive Python script based on Jupyter Notebooks for fast identification of potential spacer sequences and evaluation of off-target frequencies. The interactive structure of the Jupyter Notebooks allows simple interaction with the different intermediate and final script outputs. The script uses a FASTA file of the genome of interest as an input, and searches for PAM sites on both strands within a defined target region. Spacer sequences adjacent to those PAM sites are then extracted and their off-target frequencies calculated. One evaluation considers the entire 34 nt sequence, while the seed off-target search considers the PAM and the PAM proximal first 8 nt. Hence, seed off-targets are only counted if a PAM site is present. The resulting spacers are compiled in a pandas dataframe to allow easy filtering. Furthermore, for each spacer, the primer sequences for Gibson Assembly based cloning into pCRISPR-Cas3 are automatically generated. Given that parameters such as PAM sequence, spacer length, and seed sequence length can easily be adjusted, this script can also be used for identification of spacers for other type I CRISPR systems.

## Discussion

Here, we demonstrate the successful genome engineering of streptomycetes using a type I CRISPR system. Type I CRISPR systems are the most widespread CRISPR systems in bacteria. In *Streptomyces*, type I CRISPR systems are dominant, while type II systems are very rare. We therefore hypothesized that *Streptomyces* might be more amendable to genome engineering facilitated by type I CRISPR systems. The processive nature of the hallmark nuclease Cas3 results in long recombinogenic ssDNA overhangs. Given that *Streptomyces* have a high homologous recombination capability [61], we hypothesized that type I CRISPR systems are uniquely positioned to facilitate efficient genome engineering. The system used in this study is a previously characterized minimal type I-C CRISPR system [34].

The system, called pCRISPR-Cas3, is based on our established CRISPR platform, using a pSG5 replicon-based plasmid [62]. The CASCADE is expressed from a single promoter as a polycistronic operon. Internal RBSs facilitate successful expression of all *cas* genes. The total plasmid size (13 kb) is similar to frequently used CRISPR plasmids in *Streptomyces*, suggesting that conjugative transfer and replication in *Streptomyces* should present no problem. To overcome cloning limitations due to the need to clone spacer sequences between two CRISPR repeats, which proved to be challenging given the absence of established type IIS restriction enzymes for high GC contexts, we designed a PCR and Gibson Assembly based cloning workflow. This workflow robustly achieved high efficiencies, however limits library-based applications due to the difficulties of constructing such with PCR-based approaches. This represents a desirable improvement for future iterations of the plasmid system.

pCRISPR-Cas3 introduced highly efficient deletions of the actinorhodin BGC in *S. coelicolor*. The strong red phenotype of deletion mutants simplified screening and assessment of efficiencies. The obtained results were verified by PCR and Illumina sequencing, showing that CASCADE-Cas3 is indeed more efficient than Cas9. Previous reports of pCRISPR-Cas9 based engineering reported higher efficiencies; however, these were based on targeted screening of specific phenotypes [20]. Here, we followed an untargeted screening approach. Interestingly, successful deletions with pCRISPR-Cas3 appeared to be less dependent on the selected spacer compared to pCRISPR-Cas9. This suggests that the processive nature of the Cas3 nuclease might help to force the desired recombinations, and that Cas3 results in lower off target induced host toxicity.

The longer spacer sequence, as well as the AT rich PAM sequence, likely minimize off-target effects, thus reducing off-target associated toxicity of pCRISPR-Cas3. Direct toxicity of pCRISPR-Cas3 was only observed when spacers were used without repair templates, forcing repair through non-homologous end joining or microhomologies. Unspecific deletions were only obtained when targeting the highly plastic chromosomal arms and when targeting large chromosomal regions with many hypothetical and potentially essential genes. However, deletions extending beyond the repair templates were observed for both pCRISPR-Cas9 and pCRISPR-Cas3 and therefore resulted from targeting the highly instable chromosomal arms. By keeping this and the experimental aim in mind, pCRISPR-Cas3 can be tailored towards the desired outcome.

pCRISPR-Cas3 allowed the introduction of genomic deletions in multiple model and non-model species with high efficiencies. This suggests that pCRISPR-Cas3 can be easily implemented for genome engineering in many *Streptomyces* species. Using pCRISPR-Cas3, targeted deletions between 22 and 122 kb were achieved. While smaller deletions, such as of single genes, were not attempted in this study, given that Cas3 acts as a single-stranded DNA nuclease and hence leaves one strand intact, there is no reason why smaller deletions should not be possible. Finally, simultaneous deletions and integrations were demonstrated by substitution of entire BGCs with single PhiC31 *attB* sites. Through multiple rounds of genome engineering, the hosts *S. coelicolor* CW5 and CW6 were constructed. After BGC integration, *S. coelicolor* CW5 and CW6 produced significantly more actinorhodin compared to the base strain *S. coelicolor* CW1, resulting from the deletion of competing BGCs, a reduced genome size, as well as multicopy expression of the BGC. This BGC-*attB* substitution approach is likely to enable more streamlined construction of *Streptomyces* production hosts.

Given that pCRISPR-Cas3 is based on the established pCRISPR-Cas9 plasmid system, we hypothesize that most strains in which pCRISPR-Cas9 or CRISPR-BEST were established will also be amendable to genome engineering using pCRISPR-Cas3. Likely, pCRISPR-Cas3 will become a successor to pCRISPR-Cas9 for many applications, and enable highly efficient genome engineering, including targeted deletions, random sized deletions, as well as substitutions, even in previously difficult to engineer species. By enabling highly efficient genome engineering in more *Streptomyces* species, pCRISPR-Cas3 will likely become a crucial tool for linking BGCs to compounds, host construction, and genome reduction studies.

## Supplementary Material

gkaf214_Supplemental_File

## Data Availability

All whole genome sequencing data is available in the sequence read archive under BioProject PRJNA966932 (https://www.ncbi.nlm.nih.gov/bioproject/PRJNA966932/). Additional supplementary information such as plasmid, primer lists, and computational scripts are available at: https://figshare.com/s/e1bfb388df0ae502034e or doi: 10.11583/DTU.22786157. All plasmids, including pCRISPR-Cas3, are available on Addgene: https://www.addgene.org/Tilmann_Weber/.
